# Genome-wide CRISPR screening reveals nucleotide synthesis negatively regulates autophagy

**DOI:** 10.1016/j.jbc.2021.100780

**Published:** 2021-05-14

**Authors:** Kaito Mimura, Jun-Ichi Sakamaki, Hideaki Morishita, Masahito Kawazu, Hiroyuki Mano, Noboru Mizushima

**Affiliations:** 1 Department of Biochemistry and Molecular Biology, Graduate School and Faculty of Medicine, The University of Tokyo, Tokyo, Japan; 2 Division of Cellular Signaling, National Cancer Center Research Institute, Tokyo, Japan

**Keywords:** nucleotide, nucleoside/nucleotide biosynthesis, nucleoside/nucleotide metabolism, CRISPR/Cas, mammalian target of rapamycin, tuberous sclerosis complex, phosphoribosylformylglycinamidine synthase, BRD4, bromodomain-containing protein 4, CAD, carbamoyl-phosphate synthetase 2, aspartate transcarbamylase, and dihydroorotase, DHODH, dihydroorotate dehydrogenase, GABARAP, γ-aminobutyric acid receptor-associated protein, GeCKO, genome-scale CRISPR knockout, HEK, human embryonic kidney, LC3, microtubule-associated protein 1 light chain 3, MAGeCK, model-based analysis of genome-wide CRISPR-Cas9 knockout, mTORC1, mammalian target of rapamycin complex 1, PFAS, phosphoribosylformylglycinamidine synthase, PPAT, phosphoribosyl pyrophosphate amidotransferase, RHEB, Ras homolog enriched in brain, sgRNA, single guide RNA, TSC, tuberous sclerosis complex

## Abstract

Macroautophagy (hereafter, autophagy) is a process that directs the degradation of cytoplasmic material in lysosomes. In addition to its homeostatic roles, autophagy undergoes dynamic positive and negative regulation in response to multiple forms of cellular stress, thus enabling the survival of cells. However, the precise mechanisms of autophagy regulation are not fully understood. To identify potential negative regulators of autophagy, we performed a genome-wide CRISPR screen using the quantitative autophagic flux reporter GFP-LC3-RFP. We identified phosphoribosylformylglycinamidine synthase, a component of the *de novo* purine synthesis pathway, as one such negative regulator of autophagy. Autophagy was activated in cells lacking phosphoribosylformylglycinamidine synthase or phosphoribosyl pyrophosphate amidotransferase, another *de novo* purine synthesis enzyme, or treated with methotrexate when exogenous levels of purines were insufficient. Purine starvation-induced autophagy activation was concomitant with mammalian target of rapamycin complex 1 (mTORC1) suppression and was profoundly suppressed in cells deficient for tuberous sclerosis complex 2, which negatively regulates mTORC1 through inhibition of Ras homolog enriched in brain, suggesting that purines regulate autophagy through the tuberous sclerosis complex-Ras homolog enriched in brain-mTORC1 signaling axis. Moreover, depletion of the pyrimidine synthesis enzymes carbamoyl-phosphate synthetase 2, aspartate transcarbamylase, and dihydroorotase and dihydroorotate dehydrogenase activated autophagy as well, although mTORC1 activity was not altered by pyrimidine shortage. These results suggest a different mechanism of autophagy induction between purine and pyrimidine starvation. These findings provide novel insights into the regulation of autophagy by nucleotides and possibly the role of autophagy in nucleotide metabolism, leading to further developing anticancer strategies involving nucleotide synthesis and autophagy.

Macroautophagy (hereafter, autophagy) is an intracellular degradation system in which cytoplasmic materials are delivered to and degraded by the lysosome (1). Autophagy is responsible for the turnover of proteins and organelles and provides cells with an alternate source of nutrients for cellular renovation and homeostasis. Autophagy occurs constitutively at low levels to perform housekeeping functions, such as the degradation of dysfunctional organelles, and is upregulated in response to multiple forms of cellular stress (2), thereby serving as an essential survival mechanism.

This dynamic regulation of autophagy requires both positive and negative regulators. In addition to autophagy-related (ATG) proteins (3), many factors have been reported to positively regulate autophagy, including the endoplasmic reticulum membrane proteins VMP1 (4) and TMEM41B (5, 6, 7), proteins mediating autophagosome–lysosome fusion (*e.g.*, YKT6 (8, 9, 10), STX17 (11, 12), the HOPS complex (11, 13)), and lysosomal enzymes, which are necessary for degrading the contents of the autophagosome. Among the most important negative regulators of autophagy is mammalian target of rapamycin complex 1 (mTORC1), a central regulator of cell growth (14). mTORC1 is the master regulator of autophagy, and its suppression is the key signal for inducing autophagy. Although few in number, there have been other reports of negative regulators functioning across multiple levels within the autophagy cascade. For example, Rubicon inhibits the maturation of autophagosomes by joining the PI3KC3-C2 complex, which includes UVRAG, Beclin 1, VPS15, and VPS34 (15, 16), whereas bromodomain-containing protein 4 (BRD4) suppresses autophagy at the transcriptional level (17). A recent genome-wide screen unintentionally discovered a novel negative regulatory pathway of autophagy, mediated through ubiquitination of microtubule-associated protein 1 light chain 3 (LC3) (18).

Genome-wide CRISPR screens, first reported by two different groups in 2013 (19, 20), are displacing RNAi-based screens and have demonstrated higher efficiency and lower off-target effects (21, 22). The broad range of applications for this method includes assessing mechanisms associated with amyotrophic lateral sclerosis (23), oncology (24), immune therapy (25), and viral infections (26). In the field of autophagy research, classical RNAi-based genome-scale screens have yielded many findings (27, 28, 29, 30). In turn, CRISPR-based genome-wide screens have led to discoveries, such as the identification of the UFMylation pathway as a regulator of SQSTM1/p62 (a substrate of autophagy) (31) and ER-phagy (the autophagic degradation of the endoplasmic reticulum) (32), the discovery of an autophagy-independent lysosomal targeting pathway (33), identification of PARKIN regulators (which are the determinant of mitophagy) (34), and the identification of TMEM41B (a novel autophagy-related gene) (5, 6, 7).

Our study aimed to identify additional negative regulators of autophagy by employing a genome-wide CRISPR screen. Our nonbiased screening identified the *de novo* purine synthesis enzyme phosphoribosylformylglycinamidine synthase (PFAS) as a negative regulator of autophagy. We demonstrate in this study that purine starvation, which is caused by the complete loss of purine supply, induces autophagy activation. Our data suggest that lack of nucleotide synthesis profoundly impacts autophagy, elucidating a novel aspect of autophagy regulation.

## Results

### Genome-wide CRISPR screen for genes negatively regulating autophagy

**Figure 1:**
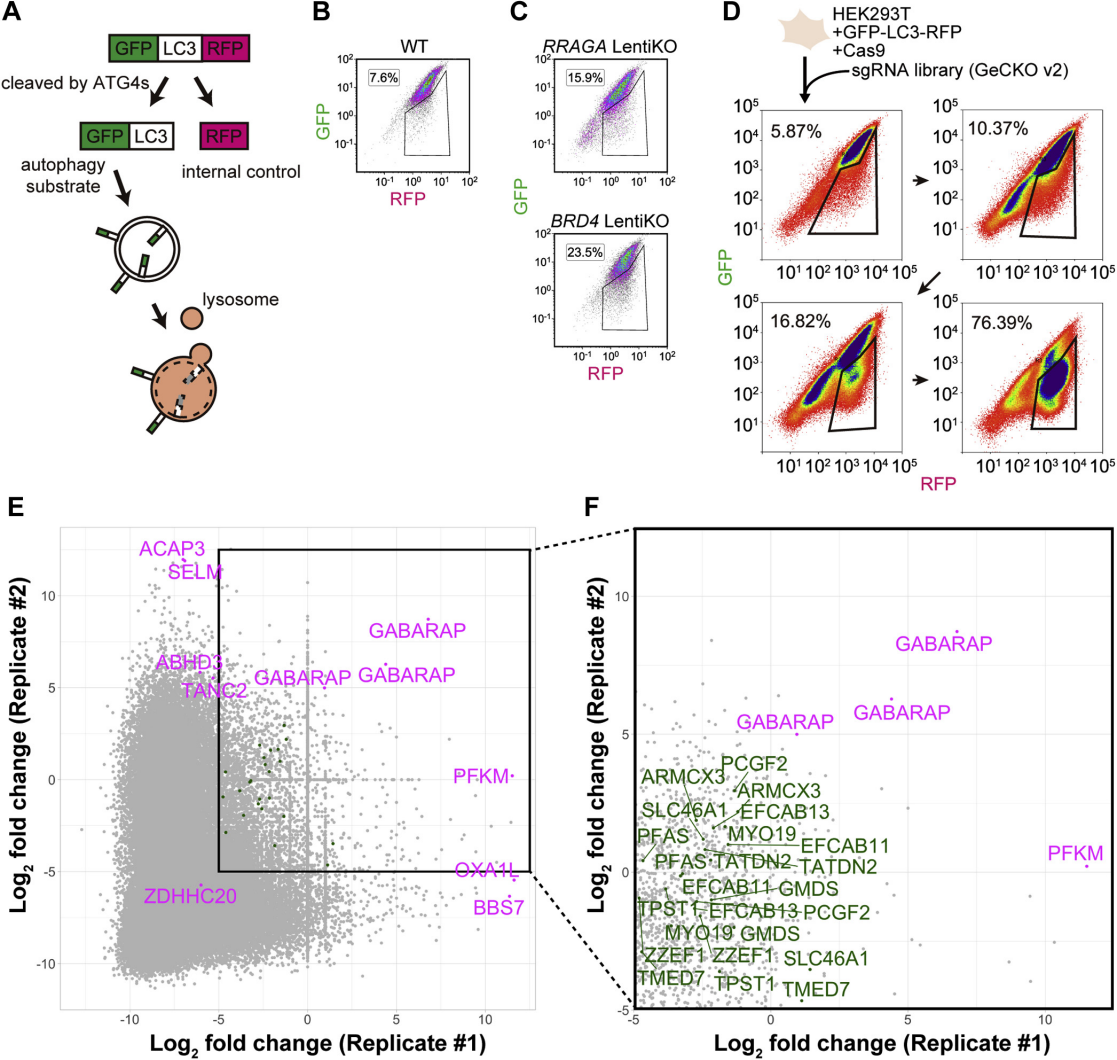
**Genome-wide CRISPR screen for genes negatively regulating autophagy.***A*, schematic representation of the GFP-LC3-RFP reporter. GFP-LC3-RFP is cleaved by endogenous ATG4 family proteases, yielding equimolar amounts of GFP-LC3 (later conjugated to autophagosomes) and RFP (released in the cytosol, serving as an internal control). *B*, GFP and RFP intensities in HEK293T GFP-LC3-RFP cells were determined by flow cytometry. The region of interest (ROI) indicates the autophagy-activated population. *C*, a LentiCRISPR-mediated bulk knockout was performed with HEK293T GFP-LC3-RFP cells using sgRNAs targeting *RRAGA* and *BRD4*. The infected cells were selected using puromycin and subjected directly to flow cytometry (without cloning). The ROI indicates the autophagy-activated population. *D*, schematic representation of the CRISPR-mediated genome-wide screen. HEK293T GFP-LC3-RFP cells were transduced with the sgRNA library (GeCKO v2) and selected with puromycin. After 2 weeks of culture, the cells were subjected to an initial sorting. The autophagy-activated population (indicated by the ROI) was sorted and cultured to expand for 2 weeks. After the fourth sorting, the sorted and unsorted (initial population) cells were subjected to next-generation sequencing. *E* and *F*, the main scatterplot of the results of two replicates (*E*). Data represent the log_2_ fold-change of read counts of each sgRNA before *versus* after enrichment. A subset scatterplot of sgRNAs that were enriched highly (>−5 log_2_ fold-change in both replicates) (*F*). In the subset scatterplot, sgRNAs with fewer than 30 read counts in the unsorted cells were filtered out. Secondary screen candidates chosen according to MAGeCK results are indicated in *magenta*, and candidates that were detected more than twice in the subset scatterplot are indicated in *green*. GeCKO, genome-scale CRISPR knockout; HEK, human embryonic kidney; LC3, microtubule-associated protein 1 light chain 3; RFP, red fluorescent protein; sgRNA, single guide RNA.

We performed a genome-wide CRISPR screen for negative regulators of autophagy. In the screen, we used the GFP-LC3-RFP autophagic flux reporter (Fig. 1*A*) (35). Endogenous ATG4 proteins, which function as proteases, cleave the GFP-LC3-RFP reporter, yielding equimolar amounts of GFP-LC3 and RFP. Subsequently, GFP-LC3 is conjugated to phosphatidylethanolamine on autophagic membranes and delivered to lysosomes, where the GFP signal is quenched. RFP stays in the cytosol and serves as an internal control. Thus, autophagic flux can be visualized by measuring the GFP:RFP ratio using flow cytometry (Fig. 1*B*).

To validate the screen, we performed a LentiCRISPR-mediated bulk knockout of a human embryonic kidney (HEK) 293T cell line stably expressing GFP-LC3-RFP and Cas9 (HEK293T GFP-LC3-RFP cells) with a LentiCRISPR vector comprising single-guide RNAs (sgRNAs) targeting previously known negative regulators of autophagy. After the introduction of sgRNAs targeting *RRAGA* (a gene coding RAGA, a positive regulator of mTORC1 (36)) and *BRD4* (a gene coding BRD4, a transcriptional repressor of autophagy (17)), the number of autophagy-activated cells with a low GFP:RFP ratio was increased (indicated by the region of interest) (Fig. 1*C*). For bulk knockouts, the phenotypes were observed according to the mutagenic efficiency of the transduced sgRNA, with limited effects on the GFP:RFP ratio of the entire population.

For the screen, HEK293T GFP-LC3-RFP cells were transduced with the human genome-scale CRISPR knockout (GeCKO) library, a pooled LentiCRISPR library comprising 123,411 sgRNAs targeting 19,050 human genes (six sgRNAs per gene) (20, 37). Infected cells were selected using puromycin. After 2 weeks of culture, the cells were sorted by flow cytometry. Cells gated in the autophagy-activated population, whose GFP signal decreased under nutrient-replete conditions, were collected. Sorting was repeated four times, at 2-week intervals (Fig. 1*D*). Genomic DNA was isolated from unsorted cells (control) and sorted cells (autophagy activated), and the sgRNA region was subjected to next-generation sequencing analysis.

We performed the screen twice and plotted the abundance of each sgRNA (Fig. 1*E* and Table S1). *GABARAP*, a gene coding the mammalian Atg8 homolog γ-aminobutyric acid receptor-associated protein (GABARAP) (38), scored highly in both replicates. This is likely because GABARAP competes with the GFP-LC3-RFP reporter to associate with autophagosomal membranes (see Discussion). Genes coding previously reported negative regulators of autophagy, such as the components of mTORC1 and BRD4, were not detected (see Discussion).

For the selection of candidate genes for the secondary screening, read counts were computationally analyzed by model-based analysis of genome-wide CRISPR-Cas9 knockout (MAGeCK) (39) (Table S2), and the top enriched genes were determined (Table S3). Additionally, based on the scatterplot, genes highly enriched in both replicates were defined as indicated in the rectangle (>−5 log_2_ fold-change in both replicates) (Fig. 1*E*). To avoid noise from low-count sgRNAs, sgRNAs with fewer than 30 read counts in the unsorted cells were filtered out (Fig. 1*F*). Genes with more than two corresponding sgRNAs remaining after processing were recorded (Table S4). From these lists, we selected a total of 21 candidate genes for the secondary screen (Fig. 1, *E* and *F*, Tables S2 and S3). Genes rarely expressed in HEK293T cells were excluded (using the published Human Protein Atlas database (40)).

### Secondary screen identifies PFAS as a novel regulator of autophagy

**Figure 2:**
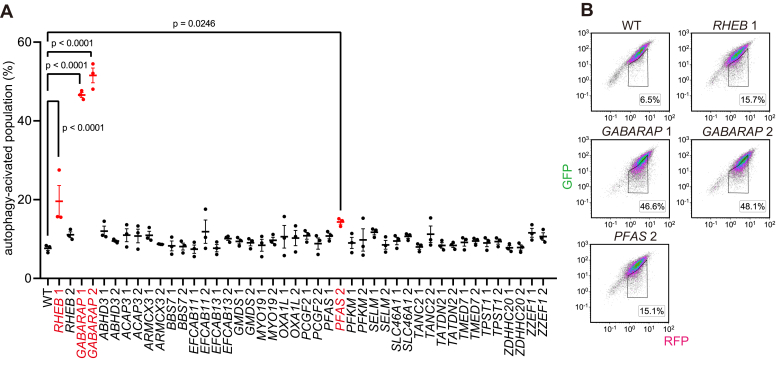
**Secondary screen identifies PFAS as a novel regulator of autophagy.***A*, results of the secondary screen. Data represent the mean ± SEM of three independent measurements. A LentiCRISPR-mediated bulk knockout was performed with HEK293T GFP-LC3-RFP cells using two independent sgRNAs for each candidate and *RHEB*. Infected cells were selected using puromycin. The ratio of the autophagy-activated population to the total population was measured using flow cytometry after at least 1 week of culture after infection. Differences were statistically analyzed by one-way ANOVA and Dunnett’s test. *B*, representative plots of cells treated with LentiCRISPR vectors targeting *GABARAP*, *RHEB*, and *PFAS* in (*A*). LC3, microtubule-associated protein 1 light chain 3; PFAS, phosphoribosylformylglycinamidine synthase; sgRNA, single guide RNA.

For the secondary screen, HEK293T GFP-LC3-RFP cells were transduced with two independent sgRNAs against each candidate gene. In addition to the 21 candidate genes, *RHEB* (an activator of mTORC1 (41)) was added as a positive control. The cells were analyzed by flow cytometry, and the ratio of the autophagy-activated population to the total population was measured (Fig. 2, *A* and *B*). A significant increase in the autophagy-activated population was observed in cells transduced with two sgRNAs for *GABARAP* and one sgRNA each for *RHEB* and *PFAS*.

### Depletion of *de novo* purine synthesis activates autophagy

**Figure 3:**
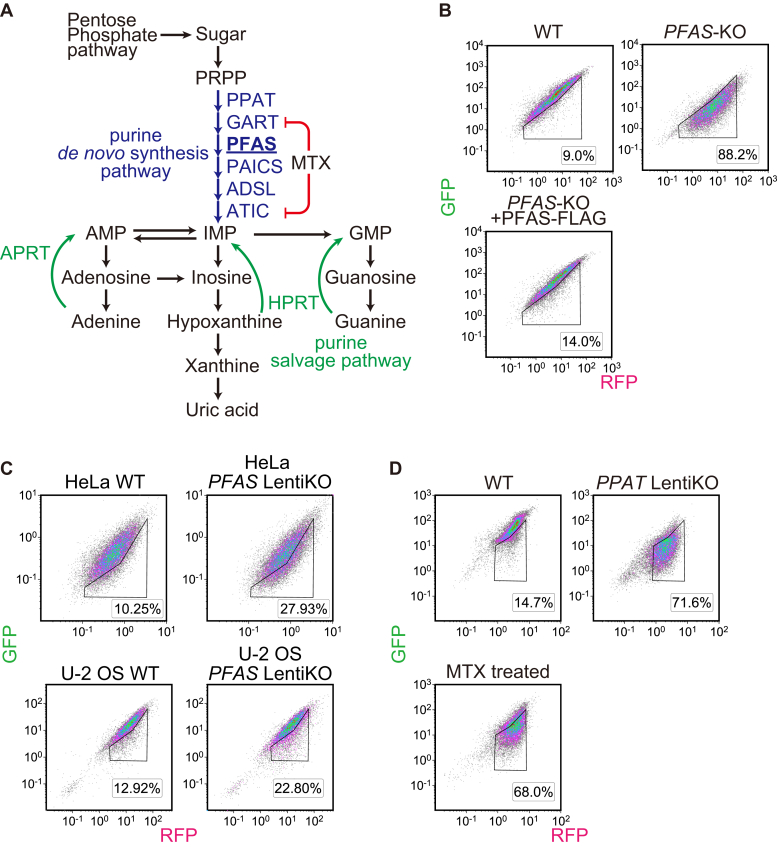
**Depletion of *de novo* purine synthesis activates autophagy.***A*, schematic representation of the purine synthesis pathways. Methotrexate (MTX) is an inhibitor of *de novo* purine synthesis. *B*, WT and *PFAS*-KO (with or without PFAS-FLAG) HEK293T cells were transduced with the GFP-LC3-RFP reporter and subjected to flow cytometry. The cells were maintained in 100 μM hypoxanthine supplemented media and then cultured without hypoxanthine supplementation for 2 days before analysis. *C*, LentiCRISPR-mediated bulk knockout using a sgRNA against PFAS was performed with HeLa and U-2 OS cells expressing the GFP-LC3-RFP reporter. These cells were maintained in 100 μM hypoxanthine-supplemented media and then cultured without hypoxanthine for 3 days before analysis. *D*, for *PPAT* Lenti KO cells, LentiCRISPR-mediated bulk knockout was performed with HEK293T GFP-LC3-RFP cells using a sgRNA against *PPAT*. Infected cells were selected using puromycin. The cells were maintained in media supplemented with 100 μM hypoxanthine and then cultured without hypoxanthine supplementation for 2 days before analysis. For MTX treatment, cells were treated with 2 μM MTX for 2 days. The autophagy-activated population is indicated by the region of interest (ROI). HEK, human embryonic kidney; LC3, microtubule-associated protein 1 light chain 3; sgRNA, single guide RNA; PFAS, phosphoribosylformylglycinamidine synthase.

*PFAS* encodes PFAS, a *de novo* purine synthesis enzyme (Fig. 3*A*). Cells depleted of PFAS show defects in *de novo* purine synthesis and are dependent on the purine salvage pathway for cell growth (42). In this study, except for in the screens, cells lacking *de novo* purine synthesis were maintained in a medium supplemented with 100 μM hypoxanthine before analysis.

To confirm the specific effect of *PFAS* knockout, we established a *PFAS*-KO HEK293T cell clone and then introduced the GFP-LC3-RFP autophagic flux reporter. When cultured in a normal culture medium (without hypoxanthine supplementation) for 2 days, the *PFAS*-KO clone showed a clear decline in the GFP:RFP signal ratio, which indicated upregulation of autophagic flux (Fig. 3*B*). This decline was restored by exogenous expression of PFAS-FLAG. These data confirm that the positive results of *PFAS* knockout in the second screen were not caused by off-target effects. In addition, LentiCRISPR-mediated bulk knockout of PFAS was performed in HeLa and U-2 OS cells expressing the GFP-LC3-RFP reporter. Autophagy activation was observed in both cell lines, suggesting that activation of autophagy by PFAS depletion is not specific to HEK293T (Fig. 3*C*)

To specify the cause of autophagy activation in PFAS-depleted cells, we performed a LentiCRISPR-mediated bulk knockout of *PPAT*, a gene encoding phosphoribosyl pyrophosphate amidotransferase (PPAT), another essential enzyme in the *de novo* purine synthesis pathway (Fig. 3*A*). Additionally, pharmacological inhibition of *de novo* purine synthesis was performed using methotrexate (Fig. 3*A*). Bulk knockout of *PPAT* and methotrexate treatment both induced autophagy activation (Fig. 3*D*). Taken together, these findings suggest that the activation of autophagy observed in *PFAS*-KO cells involves the impairment of the *de novo* purine synthesis pathway.

### Purine starvation activates autophagy

**Figure 4:**
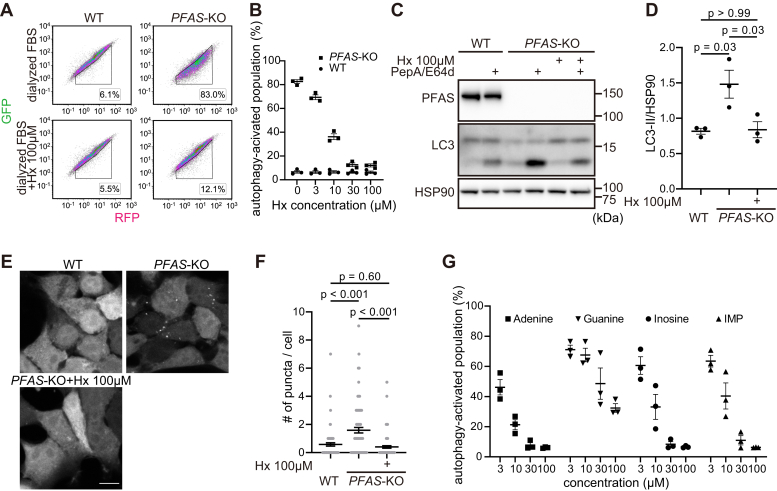
**Purine starvation activates autophagy.***A* and *B*, WT and *PFAS*-KO HEK293T cells expressing the GFP-LC3-RFP reporter were cultured for 24 h in a medium containing dialyzed FBS supplemented with or without 100 μM (*A*) or indicated concentrations of (*B*) hypoxanthine. Data represent the mean ± SEM of three independent experiments (*B*). *C* and *D*, WT and PFAS-KO (with or without 100 μM hypoxanthine [Hx]) HEK293T cells were treated for 24 h with or without lysosomal protease inhibitors pepstatin A (PepA) and E64d in a medium containing dialyzed FBS (*C*). The band intensity of LC3-II in the presence of PepA/E64d was quantified and normalized to that of HSP90. Data represent the mean ± SEM of three independent experiments. Differences were statistically analyzed by one-way ANOVA and Tukey's multiple comparison test (*D*). *E* and *F*, WT and PFAS-KO (with or without 100 μM Hx) cells expressing the GFP-LC3-RFP reporter were cultured for 24 h in a medium containing dialyzed FBS (*E*). The number of GFP-LC3 puncta per cell was statistically analyzed by one-way ANOVA and Tukey's multiple comparison test (*F*). Data were collected from >50 cells each. *G*, *PFAS*-KO HEK293T cells expressing the GFP-LC3-RFP reporter were cultured for 24 h in a medium containing dialyzed FBS supplemented with the indicated concentrations of adenine, guanine, inosine, or inosine monophosphate (IMP). Data represent the mean ± SEM of three independent experiments. HSP90, heat shock protein 90 kDa. FBS, fetal bovine serum; HEK, human embryonic kidney; LC3, microtubule-associated protein 1 light chain 3; PFAS, phosphoribosylformylglycinamidine synthase; sgRNA, single guide RNA.

Hoxhaj *et al.* (43) previously reported that when exogenous purines are depleted, cells deficient in *de novo* purine synthesis show decreased intracellular purine levels. Therefore, we investigated whether autophagy is activated in response to the depletion of extracellular purine levels in *PFAS*-KO cells. To exclude the effects of exogenous purines contained in conventional culture media containing 10% serum (estimated to be approximately 7.5 μM (44)), we used a purine-depleted medium supplemented with dialyzed serum. Activation of autophagy in PFAS-depleted cells was observed when they were cultured with less than 10 μM exogenous hypoxanthine but was canceled in the presence of sufficient hypoxanthine (more than 30 μM) (Fig. 4, *A* and *B*). Additionally, sufficient amounts of exogenous hypoxanthine suppressed the lysosomal turnover of endogenous LC3, observed by the accumulation of endogenous LC3-II upon treatment with lysosomal protease inhibitors, Pepstatin A and E64d (Fig. 4, *C* and *D*), and the formation of GFP-LC3 puncta (Fig. 4, *E* and *F*). Given that cells deficient in *de novo* purine synthesis are dependent on the salvage pathway, *PFAS*-KO cells cultured in a purine-insufficient medium should have been subjected to intracellular purine-starvation conditions. These data suggest that the decline of intracellular purine levels, caused by purine starvation, activates autophagy.

Aside from hypoxanthine, sufficient amounts (more than 30 μM) of exogenous inosine, inosine monophosphate, and adenine could completely cancel the autophagy activation, whereas the effect of guanine was limited (Fig. 4*G*). Together with our findings on purine synthesis pathways (Fig. 3*A*), the unavailability of hypoxanthine or adenine and their derivatives could mediate purine starvation-induced autophagy.

### Purine starvation activates autophagy through the tuberous sclerosis complex-Ras homolog enriched in brain-mTORC1 signaling axis

**Figure 5:**
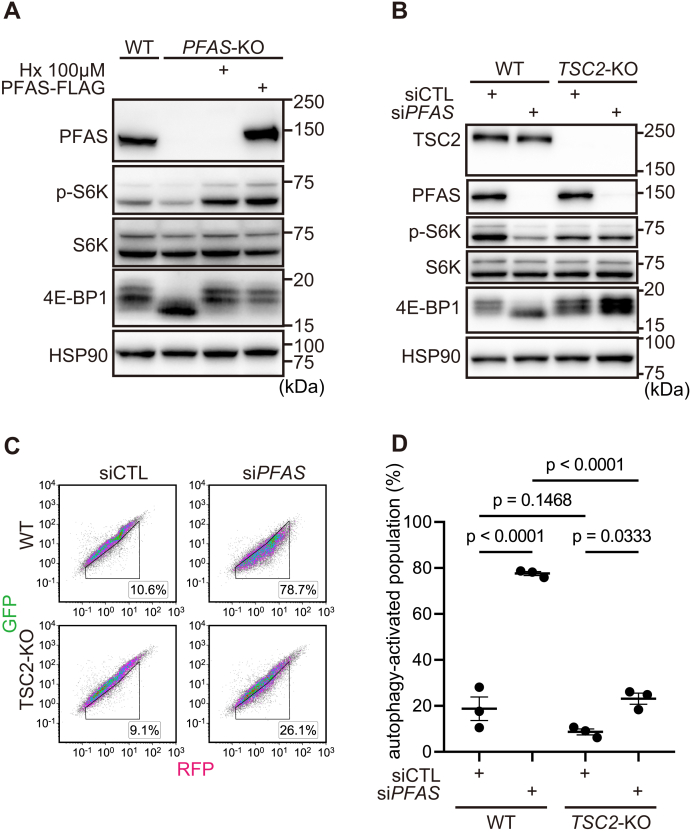
**Purine starvation activates autophagy through the TSC-RHEB-mTORC1 signaling axis.***A*, WT and *PFAS*-KO (with or without PFAS-FLAG) HEK293T cells were cultured for 24 h in a medium containing dialyzed FBS. *PFAS*-KO cells were also treated with 100 μM hypoxanthine. *B*, WT and *TSC2*-KO HEK293T cells were transfected with negative control siRNA (siCTL) or siRNA targeting *PFAS* and cultured for 24 h in a medium containing dialyzed FBS. *C* and *D*, WT and *TSC2*-KO HEK293T cells expressing the GFP-LC3-RFP reporter were transfected with negative siCTL or siRNA targeting *PFAS* and cultured for 24 h in a medium containing dialyzed FBS. The ratio of the autophagy-activated population was quantified (*C*). Data represent the mean ± SEM of three independent experiments. Differences were statistically analyzed by one-way ANOVA and Tukey's multiple comparison test (*D*). FBS, fetal bovine serum; HEK, human embryonic kidney; LC3, microtubule-associated protein 1 light chain 3; mTORC1, mammalian target of rapamycin complex 1; PFAS, phosphoribosylformylglycinamidine synthase; RHEB, Ras homolog enriched in brain; sgRNA, single guide RNA; TSC, tuberous sclerosis complex.

Next, we investigated the mechanism of autophagy activation in purine-starved cells. Recent studies have shown that depletion of intracellular purine bases *via* inhibition of purine synthesis pathways suppresses mTORC1 (43, 45). Therefore, we investigated whether the activation of autophagy is concomitant with the downregulation of mTORC1 in PFAS-depleted cells. Consistent with previous studies, mTORC1 activity was downregulated in *PFAS*-KO cells depleted of purines, as assessed from the phosphorylation status of its substrates p70 S6-kinase and eukaryotic translation initiation factor 4E-binding protein (Fig. 5*A*). mTORC1 activity was restored by hypoxanthine treatment or expression of PFAS–FLAG.

Previous studies have suggested that purines regulate mTORC1 in a manner dependent on the tuberous sclerosis complex (TSC) protein complex and Ras homolog enriched in brain (RHEB) (43, 45). To investigate whether autophagy is also regulated by this TSC-RHEB signaling axis, we generated a *TSC2*-KO HEK293T cell clone and depleted PFAS using a siRNA targeting *PFAS*. In *TSC2*-KO cells, silencing of PFAS did not inhibit mTORC1 activity even under purine starvation conditions (Fig. 5*B*). As assessed by the GFP-LC3-RFP reporter assay (Fig. 5, *C* and *D*), the activation of autophagy was profoundly suppressed in *TSC2*-KO cells under purine starvation conditions. These findings suggest that purine starvation induces autophagy primarily through the TSC-RHEB-mTORC1 signaling axis.

### Depletion of pyrimidine *de novo* synthesis enzymes activates autophagy

**Figure 6:**
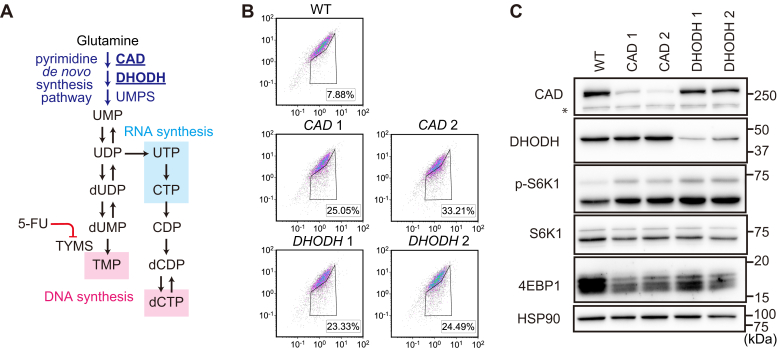
**Depletion of pyrimidine *de novo* synthesis enzymes activates autophagy.***A*, schematic representation of the pyrimidine synthesis pathways. *B*, a LentiCRISPR-mediated bulk knockout was performed with GFP-LC3-RFP-expressing HEK293T cells using two independent sgRNAs, each for *CAD* and *DHODH*. Infected cells were selected using puromycin. The ratio of the autophagy-activated population to the total population was measured using flow cytometry after at least 1 week of culture after infection. *C*, immunoblotting of LentiCRISPR-mediated bulk knockout HEK293T GFP-LC3-RFP cells. 5-FU, 5-fluorouracil; CAD, carbamoyl-phosphate synthetase 2, aspartate transcarbamylase, and dihydroorotase; DHODH, dihydroorotate dehydrogenase; HEK, human embryonic kidney; LC3, microtubule-associated protein 1 light chain 3; sgRNA, single guide RNA; TYMS, thymidylate synthase.

We also determined whether a blockade of pyrimidine synthesis also affects autophagy. We performed LentiCRISPR-mediated bulk knockout of *CAD* and *DHODH*, genes which code enzymes involved in *de novo* pyrimidine synthesis: carbamoyl-phosphate synthetase 2, aspartate transcarbamylase, and dihydroorotase (CAD), and dihydroorotate dehydrogenase (DHODH) (Fig. 6*A*). When the cells were cultured with dialyzed serum, bulk knockouts of *CAD*, or *DHODH* resulted in autophagy activation (Fig. 6*B*) but without mTORC1 inactivation (Fig. 6*C*). These results suggest that pyrimidine starvation also activates autophagy, but likely in an mTORC1-independent manner.

## Discussion

By conducting a genome-wide CRISPR screen, we identified PFAS as a novel negative regulator of autophagy. Autophagy activation in PFAS-KO cells was confirmed by measuring the lysosomal turnover of endogenous LC3 and quantifying the number of GFP-LC3 puncta. Further investigation of *PFAS*-KO cells revealed that purine starvation, which is caused by inhibition of both the *de novo* purine synthesis pathway and the salvage pathway, activates autophagy *via* the TSC-RHEB-mTORC1 signaling axis. Finally, we show that pyrimidine shortage also activates autophagy in an mTORC1-independent manner.

Our screen yielded only a few hits, suggesting that there are not many robust negative regulators of autophagy. We did not identify previously reported negative regulators of autophagy, such as the components of mTORC1, in this screen. Given that the rate of cell growth biases the results of this screen, genes required for cell growth or survival cannot be enriched. This seems to be the case for mTORC1 and BRD4 (46, 47). In addition, excess autophagy may compromise cell growth. The use of an inducible CRISPRi strategy could avoid the effect of growth restriction caused by gene suppression during the expansion of sorted cells (48).

We observed *PFAS*-KO cells to be auxotrophic for extracellular purines, showing growth restriction in normal culture conditions after 1 to 2 days of proliferation. In contrast, *PFAS*-KO cells cultured in dialyzed-serum supplemented media (depleted of purines) exhibited immediate growth restriction. The exogenous purine concentration in normal culture media containing 10% serum (estimated to be approximately 7.5 μM (44)) was sufficient for only a short period of culture and may have been eventually depleted after 1 to 2 days. Nevertheless, *PFAS*-KO cells survived the screen, with two sgRNAs enriched in both replicates. This may be because most other cells preferentially used the *de novo* purine synthesis pathway for growth (49, 50). Consequently, the extracellular purines present in normal culture medium may not have been critically depleted, permitting the small population of *PFAS*-KO cells to proliferate by using exogenous purines during the screen. However, the reason that the other *de novo* purine synthesis enzymes were not detected in the screen remains elusive.

*GABARAP* was by far the highest hit gene in our screen. GABARAP is a mammalian homolog of yeast Atg8, as well as LC3A, LC3B, LC3C, GABARAPL1, and GABARAPL2. It can therefore be hypothesized that LC3 and GABARAP family proteins compete with each other for localization to autophagosomes. In the absence of GABARAP, the recruitment of the GFP-LC3-RFP reporter to autophagosomes may have increased, resulting in boosted degradation and a decrease in the GFP:RFP ratio. The reason that no mammalian ATG8 proteins other than GABARAP were detected remains elusive; one possible explanation is that GABARAP is the highest expressed ATG8 protein in HEK293T cells (40), and it may thus occupy a large area on the autophagosome. This phenomenon of competitive inhibition may apply to any autophagic flux assay using mammalian ATG8 proteins.

The upstream signaling of the TSC-RHEB-mTORC1 complex in purine starvation conditions remains elusive. A previous study using pharmacological inhibitors of *de novo* purine synthesis suggests that adenylate or its derivatives such as ATP mediate the mTORC1-suppressive effect of purines in an AMPK-independent manner (43). Another study, using pharmacological inhibitors as well, suggests that guanine nucleotide depletion may diminish GTP-bound RHEB, leading to mTORC1 inactivation (45). However, the exact metabolite required and the precise sensing mechanism of purine starvation remain unknown and await further investigation. Studying the upstream factors in *PFAS*-KO cells, in which *de novo* purine synthesis is impaired genetically, should be important.

The physiological importance of the response of autophagy to purine or pyrimidine nucleobase starvation may be that cells detect a shortage of nucleobases and attempt to compensate through the degradation of cellular components. A recent study discussed that degradation of ribosomal RNAs *via* autophagy is crucial for maintaining cellular pyrimidine pools during *C. elegans* development (51), suggesting that autophagy may contribute to the maintenance of cellular nucleobase pools. In lung cancer cells, autophagy is essential for maintaining cellular nucleotide pools (52). Given that nucleotides are *de novo* synthesized using amino acids, autophagy may provide amino acids such as glutamate and aspartate as substrates, as a recent study reports (53). Another study reported that yeast cells under nitrogen starvation conditions, under which cells cannot proliferate, degrade cellular RNA by autophagy into purine and pyrimidine bases, which is eventually excreted from the cell without being reused (54). In our study, we observed that extracellular nucleobases could rescue cells under purine or pyrimidine starvation, and although, cells may primarily excrete RNA degradation products, reuptake, and salvage may occur.

Our results suggest that the shortage of cellular pyrimidines activates autophagy in an mTORC1-independent manner, which is different from that of purines. This may be consistent with previous findings that 5-fluorouracil, a pyrimidine derivative that inhibits thymidylate synthase (Fig. 6*A*), activates autophagy (55, 56, 57, 58). Although the exact molecular mechanism remains poorly understood, previous studies demonstrated that inhibition of autophagy augments the anticancer effects of 5-fluorouracil (58, 59). Revealing the precise mechanism of autophagy induction by inhibition of pyrimidine synthesis may contribute to further developing such anticancer strategies.

Selective autophagy—especially ribophagy, which is a selective turnover of ribosomes by autophagy—may be a source of purine bases as well (60). NUFIP1 is reported to be a receptor for starvation-induced ribophagy (61). However, another recent study suggests that ribosomal proteins are turned over mainly through nonautophagic pathways (62), which contradicts the autophagic turnover of ribosomes. Further research on purine-starved cells would provide new insights into the regulation of autophagy by nucleic acids and its physiological significance.

## Experimental procedures

### Antibodies and reagents

For immunoblotting, the following antibodies were used: a mouse monoclonal antibody against heat shock protein 90 kDa (610419; BD Transduction Lab) and rabbit polyclonal antibodies against PFAS (A17517; Abclonal), 4E-binding protein 1 (9452; Cell Signaling Technology), p70 S6 Kinase (9202; Cell Signaling Technology), phospho-p70 S6 Kinase (T389) (9234; Cell Signaling Technology), LC3 (63), TSC2 (3990; Cell Signaling Technology), CAD (11933; Cell Signaling Technology), and DHODH (14877-1-AP; Proteintech). For secondary antibodies, horseradish peroxidase–conjugated anti-mouse (111-035-003; Jackson ImmunoResearch Laboratories, Inc) and anti-rabbit (111-035-144; Jackson ImmunoResearch Laboratories, Inc) IgGs were used.

### Plasmids

A HEK293T cell line stably expressing GFP-LC3-RFP and Cas9 (HEK293T GFP-LC3-RFP cells) was generated as previously described (6). LentiCas9-Blast (52962 (37); Addgene), human GeCKO v2 Library (1000000049 (37); Addgene), lentiCRISPR v2 (52961 (37); Addgene), and pSp-Cas9(BB)-2A-Puro (48139 (64); Addgene) were provided by F. Zhang (Massachusetts Institute of Technology, Cambridge, MA). For CRISPR knockouts, sgRNAs were cloned into lentiCRISPR v2 and pSp-Cas9(BB)-2A-Puro, sequences of which are listed in Table S5. For stable expression, GFP-LC3-RFP reporter was inserted into pMRX-IP (65) and pMRX-IB (6), and *PFAS* (NM_012393) tagged with 3 × FLAG was inserted into pMRX-IB (6) (these plasmids were generated from pMXs (66)).

### Cell lines

HEK293T cells were cultured in Dulbecco's modified Eagle's Medium (D6546; Sigma-Aldrich) supplemented with 10% fetal bovine serum (FBS) (S1820-500; Biowest/173012; Sigma-Aldrich), 50 U/ml penicillin, 50 μg/ml streptomycin (15070-063; GIBCO), and 2 mM glutamine (25030-081; GIBCO) in a 5% CO_2_ incubator (hereafter referred to as normal culture conditions). For purine depletion experiments, cells were cultured in purine-depleted media, which is Dulbecco's modified Eagle's Medium supplemented with 10% dialyzed FBS (SH30079; Hyclone), 50 U/ml penicillin, 50 μg/ml streptomycin, and 2 mM glutamine. In the indicated experiments, cells were cultured with 2 μM methotrexate (135-13573; Wako), 10 μg/ml pepstatin A (4397-v; Peptide Institute), 10 μg/ml E64d (4321-v; Peptide Institute), hypoxanthine (086-03403; Wako), adenine (A0149; Tokyo Chemical Industry), guanine (G0169; Tokyo Chemical Industry), inosine (I4125; Sigma-Aldrich), or inosine 5'-monophosphate disodium salt hydrate (I0036; Tokyo Chemical Industry). Except for the screen, cells lacking *de novo* purine synthesis were maintained in media supplemented with 100 μM hypoxanthine, and the media were changed before analysis.

### Generation of stable cell lines

Lentivirus was prepared using HEK293T cells transiently transfected with a lentiviral vector together with psPAX2 (a gift from D. Trono, Ecole Polytechnique Federale de Lausanne) and pCMV-VSV-G (a gift from R.A. Weinberg, Whitehead Institute for Biomedical Research). After 2 days of culture, the supernatant was passed through a 0.45-μm syringe filter unit (SLHV033RB; EMD Millipore). For retrovirus transfection, HEK293T cells were transiently transfected with a retroviral vector together with pCG-VSV-G and pCG-gag-pol (gifts from T. Yasui, Osaka University). As described above, the virus was collected from the supernatant. Transfections of plasmids were all performed using Lipofectamine 2000 (11668019; Thermo Fisher Scientific) according to the manufacturer's instructions. After cells were infected with retrovirus or lentivirus, stable transformants were selected with puromycin (P8833; Sigma-Aldrich).

### RNAi

Silencer Select negative control No. 1 siRNA (4390843; Thermo Fisher Scientific) and PFAS siRNA (4392420 (s10330); Thermo Fisher Scientific) were transfected into cells using Lipofectamine RNAiMAX (13778150; Thermo Fisher Scientific) according to the manufacturer's instructions. The medium was replaced 24 h after transfection. The cells were cultured for an additional 2 days before analysis.

### Genome-wide CRISPR screen

HEK293T cells expressing the GFP-LC3-RFP probe and Cas9 were infected with the pooled lentiviral library at a multiplicity of infection of 0.3 to 0.5 and selected with puromycin. Two weeks after infection, the cells were subjected to flow cytometric cell sorting performed as follows: cells were resuspended with Hanks' balanced salt solution (H6648; Sigma-Aldrich) containing 10% FBS, penicillin, and streptomycin and were then sorted by flow cytometry (MoFlo AstriosEQ; Beckman Coulter). After the cells were sorted, the cells were cultured and expanded for roughly 2 weeks and then sorted again by flow cytometry. Enrichment by flow cytometric cell sorting was repeated four times.

### Genomic DNA sequencing

As previously described (20), genomic DNA was sequenced, and data were processed. First, 4 × 10^7^ cells (*i.e*., >300× coverage over the GeCKO v2 library) were collected for genomic DNA extraction, using the Blood & Cell Culture Midi kit (13343; Qiagen). A total of 100 to 240 μg of genomic DNA (varying between replicates) were extracted, and sgRNA sequences were amplified by PCR using Herculase II Fusion DNA polymerase (600675; Agilent Technologies). Next, the amplicons were subjected to a second PCR amplification to attach Illumina adaptors and barcode samples. The primers used in the first PCR were as follows: F1, 5'-AATGGACTATCATATGCTTACCGTAACTTGAAAGTATTTCG-3’; R1, 5’-CTTTAGTTTGTATGTCTGTTGCTATTATGTCTACTATTCTTTCC-3’. The primers used in the second PCR were as follows: F2, 5’-AATGATACGGCGACCACCGAGATCTACACTCTTTCCCTACACGACGCTCTTCCGATCT-(variable-length sequence, up to 8 bp)-TCTTGTGGAAAGGACGAAACACCG-3’; R2, 5’-CAAGCAGAAGACGGCATACGAGAT-(8-bp barcode)-GTGACTGGAGTTCAGACGTGTGCTCTTCCGATCTTCTACTATTCTTTCCCCTGCACTGT-3’. DNA was sequenced on a HiSeq 2500 (Illumina) sequencer in rapid run mode, with a single-ended read length of 71 bp. After data acquisition, the number of reads that completely matched the GeCKO v2 library sequence was calculated.

### Analysis of next-generation sequencing data

Scatter plots were generated in the R statistical computing environment using the ggplot2 data visualization package. For the generation of the main scatter plot, a pseudo-read count of 0.5 was applied to all read counts to avoid dividing by 0 and requiring the calculation of the logarithm of 0. For the subset scatter plot, a pseudo read count of 0.5 was applied, and sgRNAs with fewer than 30 reads in the control cells for either replicate were omitted; sgRNAs with log_2_ fold-changes >−5 were plotted.

MAGeCK (v0.5.9.2) was used for the analysis of read counts (39). All parameters except for the following were set as default: paired sample comparison; zero-count sgRNAs removed from control reads; normalization using total read counts; sorting criteria set to positive selection.

Gene expression data were obtained from the Human Protein Atlas (http://www.proteinatlas.org) (40).

### Flow cytometry

Cells were treated with trypsin-EDTA (25300062; GIBCO) for 2 min and collected in ice-cold PBS. The cells were washed once, resuspended in ice-cold PBS, and analyzed on an EC800 cell analyzer (Sony Japan) equipped with 488-nm and 561-nm lasers. Data were processed using Kaluza software (Beckman Coulter).

### Immunoblotting

Cells were lysed using a lysis buffer (50 mM Tris-HCl, pH 7.5 or 25 mM Hepes, pH 7.2, 150 mM NaCl, 2 mM EDTA, 1% Triton X-100, and Protease inhibitor cocktail [EDTA free] (03969; Nacalai)) for 15 min on ice and then centrifuged at 12,000*g* for 15 min. The supernatant was collected and boiled in sample buffer (46.7 mM Tris-HCl, pH 6.8, 5% glycerol, 1.67% sodium dodecyl sulfate, 1.55% dithiothreitol, and 0.02% bromophenol blue). Samples were separated by SDS-PAGE and transferred to Immobilon-P polyvinylidene difluoride membranes (IPVH00010; EMD Millipore). The antibodies used are described above. Immobilon Western Chemiluminescent horseradish peroxidase Substrate (P90715; EMD Millipore Corporation) or Super-Signal West Pico Chemiluminescent Substrate (1856135; Thermo Fisher Scientific) was used to visualize the signals. The signals were detected on a Fusion Solo 7S system (M&S Instruments Inc). Images were adjusted using Photoshop CC 2018 (Adobe). Quantitation of bands was performed using Fiji (67).

### Fluorescence microscopy

Cells grown on coverslips were washed with PBS and fixed with 4% paraformaldehyde in PBS (09154-85; Nacalai) for 15 min at room temperature. Fixed cells were stained with 1 μg/ml Cellstain Hoechst 33342 (23491-52-3; Wako) in Hanks' balanced salt solution for 10 min. After washing twice with PBS, the coverslips were mounted in Prolong Gold antifade reagent (P36930; Thermo Fisher Scientific). The coverslips were observed using a confocal laser microscope (FV1000 IX81; Olympus) with a 60× oil-immersion objective lens (PLAPON60XO; Olympus) and captured with Fluoview software (Olympus). Contrast and brightness adjustment was performed using Fiji (67). The number of punctate structures was determined using Cellprofiler (68).

### Statistical analysis

Multiple comparisons were performed by one-way analysis of variance followed by Dunnett’s test or Tukey's multiple comparison test using GraphPad Prism 8 (GraphPad Software). Normal distributions were assumed but not formally tested. Statistical significance was determined at a *p* < 0.05 threshold.

## Data availability

Raw sequencing reads and results of analysis by MAGeCK are available online at NCBI GEO (GSE164778). Any additional data supporting the analyses in the manuscript are available from the corresponding author upon reasonable request.

## Supporting information

This article contains supporting information.

## Conflict of interest

The authors declare that they have no conflicts of interest with the contents of this article.
