# Supplementary material for: Genome-wide CRISPR screening reveals nucleotide synthesis negatively regulates autophagy
Source: J Biol Chem. 2021 May 14;296:100780. doi: 10.1016/j.jbc.2021.100780 (PMC8191307; doi:10.1016/j.jbc.2021.100780)
Supplement: Supplemental Tables S3–S5 [file mmc3.pdf]

Supporting information

Genome-wide CRISPR screening reveals autophagy regulation by purines

Kaito Mimura[1], Jun-ichi Sakamaki[1], Hideaki Morishita[1§], Masahito Kawazu[2], Hiroyuki Mano[2], Noboru Mizushima[1¶]

[1]Department of Biochemistry and Molecular Biology, Graduate School and Faculty of Medicine, The University of Tokyo, Tokyo 113-0033, Japan
[2]Division of Cellular Signaling, National Cancer Center Research Institute, Tokyo, Japan

[§]Current address: Department of Physiology, Juntendo University Graduate School of Medicine, Tokyo 113-8421, Japan

[¶]Corresponding author: Noboru Mizushima
E-mail: nmizu@m.u-tokyo.ac.jp
Running title: Autophagy regulation by purines

Supplementary Table S1: Read counts of next-generation sequencing
Supplementary Table S2: Gene list analyzed by MAGeCK

Supplementary Table S3: Top enriched genes calculated by MAGeCK

	gene	*p*-value	FDR*	2nd screening
1	*GABARAP*	2.28E-07	0.00495	yes
2	*PFKM*	7.24E-05	0.784653	yes
3	*OXA1L*	0.0002326	0.94066	yes
4	*PLXNC1*	0.0002586	0.94066	
5	*BBS7*	0.0004079	0.94066	yes
6	*hsa-mir-4714*	0.0004038	0.94066	
7	*PRKCA*	0.0005769	0.94066	
8	*hsa-mir-3137*	0.0004349	0.94066	
9	*AFP*	0.0006522	0.94066	
10	*C1orf185*	0.0006577	0.94066	
11	*SELM*	0.0007476	0.94066	yes
12	*ACAP3*	0.0007841	0.94066	yes
13	*ABHD3*	0.000844	0.94066	yes
14	*TANC2*	0.0007321	0.94066	yes
15	*EPB41L3*	0.0009421	0.94066	
16	*SUPT6H*	0.000749	0.94066	
17	*ZDHHC20*	0.0009645	0.94066	yes

*false discovery rate

Supplementary Table S4: List of genes for which corresponding sgRNAs were detected more than twice in the subset scatterplot (Fig. 1 F)

gene	2nd screening
GABARAP	yes
ABP1	
ARMCX3	yes
COX4I2	
EFCAB11	yes
EFCAB13	yes
FAM181A	
GAGE1	
GMDS	yes
GRM3	
hsa-mir-642a	
IRGC	
MYO19	yes
NMT2	
OR13C3	
OR2L8	
OR51I2	
PABPC1L2A	
PCGF2	yes
PFAS	yes
PRB3	
PRR15L	
RBMY1A1	
SLC46A1	yes
TATDN2	yes
TCEAL2	
TMED7	yes
TPST1	yes
ZZEF1	yes

Supplementary Table S5: Sequences of sgRNAs cloned into CRISPR vectors

vector	target	sequence
lentiCRISPR v2	*RRAGA*	5'-GGAGTGTTCCACGTCAATGG-3'
	BRD4	5'-TTCAGCTTGACGGCATCCAC-3'
	PPAT	5'-AGACCAGACAGTATGTTCGA-3'
	RHEB 1	5'-CTTCAACTTGTAGACACAGC-3'
	RHEB 2	5'-TTATGAAGAAGGGAAAGCTT-3'
	ABHD3 1	5'-CCGAGCCGAAGAACCCCACC-3'
	ABHD3 2	5'-AGCGAAGGTGTTCCAACCAA-3'
	ACAP3 1	5'-GGTAGTTCACCACCTCCTGT-3'
	ACAP3 2	5'-TAGGGCGACCATTGACGAGG-3'
	ARMCX3 1	5'-TCACCGAATTGATTCTGAGT-3'
	ARMCX3 2	5'-ACGATTGCATGGGCTACGCC-3'
	BBS7 1	5'-AAGACACAGAGCTACACAAA-3'
	BBS7 2	5'-TACCCGGGCCGAAGATTGCA-3'
	EFCAB11 1	5'-TTCATATTACCAGAAGTATT-3'
	EFCAB11 2	5'-GTTTGGGTACAAGCCCTCCA-3'
	EFCAB13 1	5'-TCCTTTGACAGCTTCAGAAA-3'
	EFCAB13 2	5'-TTGTGAACGCTGTACTGATT-3'
	GABARAP 1	5'-GTAGACACTTTCGTCACTGT-3'
	GABARAP 2	5'-TTCGAGAAGCGCCGCTCTGA-3'
	GMDS 1	5'-TCCCCAGGATGGTTCCTACC-3'
	GMDS 2	5'-TGGACCGCCGTACAATTCCA-3'
	MYO19 1	5'-CTGGAGCCTCTCGTCCTCAC-3'
	MYO19 2	5'-ACAACAATAGACTGGTTGAC-3'
	OXA1L 1	5'-GCTCAGGTCCACAGCGTCGC-3'
	OXA1L 2	5'-GGGACTAATGTGCGGACGCC-3'
	PCGF2 1	5'-CAGATACCTTTAAAAAGCCC-3'
	PCGF2 2	5'-CATCGACGCCACCACTATCG-3'
	PFAS 1	5'-AGCAACATCATCCAGCAGTA-3'
	PFAS 2	5'-TCAACCCCACATACCTTCCG-3'
	PFKM 1	5'-TTCAGTCTCGCCAGTTAGTC-3'

	PFKM 2	5'-CCTACAACCTGGTGAAGCGT-3'
	SELM 1	5'-CTAGCGCATTGATCTCTTCG-3'
	SELM 2	5'-CGGCGTACTCACCTCTACCC-3'
	SLC46A1 1	5'-GGCTGCTAGCTTTGCGTCCG-3'
	SLC46A1 2	5'-GCGGTGCCACAGATACTGCG-3'
	TANC2 1	5'-TTCATCTACTCGATACAAAC-3'
	TANC2 2	5'-CTTGACTGTCTTCGATCCGG-3'
	TATDN2 1	5'-GCGTCCTGCCAACGCCTCTT-3'
	TATDN2 2	5'-ACGATGTGGCTTGCTCGCGG-3'
	TMED7 1	5'-CTCTTACAGGTGATTACTGG-3'
	TMED7 2	5'-GCACCCCCAACGGCCCGCGA-3'
	TPST1 1	5'-GCCATGTCTTGTAAAACATC-3'
	TPST1 2	5'-TACGTTCCTCTATCCGGTGA-3'
	ZDHHC20 1	5'-ATTAAATTTACCTCTTTGGA-3'
	ZDHHC20 2	5'-GAGTAACACTTACGAGTCAC-3'
	ZZEF1 1	5'-CTTGTCAATGTCTGCCGAGT-3'
	ZZEF1 2	5'-CTCTAGCGAGCAGTTCGAGG-3'
	CAD 1	5'-CCACGGGTATTCAATACAGG-3'
	CAD 2	5'-GGATGAGTTCGGTCTCTGCA-3'
	DHODH 1	5'-ATAGAAACGCTCATCTCCCG-3'
	DHODH 2	5'-CATAAATTCCGAAATCCAGT-3'
pSp-Cas9(BB)-2A-Puro	*PFAS*	5'-TCAACCCCACATACCTTCCG-3'
	TSC2	5'-AGTATCTCTCCTGAGACCCG-3'
